# Diabetic patients are at a higher risk of lacunar infarction and dyslipidemia: results of a comparative pilot study from King Fahad Hospital of the University, Saudi Arabia

**DOI:** 10.17712/nsj.2017.1.20160302

**Published:** 2017-01

**Authors:** Azra Zafar

**Affiliations:** *From the Department of Neurology, Assistant Professor, Consultant Neurologist, King Fahad Hospital of the University, Alkhobar, Kingdom of Saudi Arabia*

## Abstract

**Objective:**

To describe the various risk factors and identify the characteristics of ischemic stroke in diabetic patients.

**Method:**

A retrospective study carried out at King Fahd Hospital of the University in Al-Khobar, kingdom of Saudi Arabia from February 2010 to December 2015. Statistical analysis was performed by the Statistical Package for the Social Sciences (version 22.0, SPSS Inc, Chicago, IL, USA).

**Results:**

One hundred and twenty-seven diabetic patients with ischemic stroke were compared with 127 non-diabetics. Mean age was 61±13.6 (mean±SD) years for diabetics and 60±16.6 years for non-diabetics. There were 68.5% male in the diabetic group and 62.2% in the non-diabetic group. Small vessel occlusion was the most common subtype (41.7%) in diabetics and stroke of undetermined etiology (32%) in non-diabetics. Dyslipidemia was significantly more prevalent in diabetics as compared with non-diabetics.

**Conclusion:**

Our study found significant differences in characteristics of ischemic stroke in diabetics compared with non-diabetics with dyslipidemia, microangiopathy, and lacunar infarction being more frequent. Further epidemiological studies are required to understand the characteristics of strokes in diabetics.

Approximately 16 million first ever strokes occur in the world annually, causing a total of 5.7 million deaths.^1^ Ischemic strokes (IS) account for about 87% of all strokes.^2^ Diabetes mellitus is one of the major modifiable risk factors for IS. Patients with diabetes are at least 2 times more likely to have a stroke than non-diabetics.^3^ A high prevalence of stroke has been identified not only in patients with diagnosed and undiagnosed diabetes but also with glucose intolerance.^4^ Subjects with diabetes and elevated glucose are said to be at increased risk of ischemic cerebral infarcts^3^,^5^, especially lacunar infarcts^4^ but not cerebral hemorrhage.^3^-^5^ Data accumulating over the last 3 decades have confirmed that there is an epidemic of type 2 diabetes in Kingdom of Saudi Arabia (KSA). It was suggested that this has resulted from significant socioeconomic transformation and change in life style.^6^ Studies from KSA have reported diabetes in 37-41% of patients with stroke.^7^,^8^ Diabetic patients with stroke have some features distinctive from their non diabetic counterparts. There is conflicting data in published literature about it. Overall, lacunar stroke is said to be more prevalent in diabetics as compared with non-diabetics.^4^,^9^,^10^ One study from KSA found young Saudi diabetic patients to be more likely to have lacunar infarctions.^11^ Among patients with diabetes, several risk factors contribute to the development of IS. Hyperglycemia and vascular risk factors such as hypertension and dyslipidemia are considered as diabetes-specific factors.^12^ However, genetics, demographics and lifestyle factors also contribute to variable degree.^13^ There is a lack of published literature on ISs in diabetic patients in Kingdom of Saudi Arabia (KSA). The aim of this study is to describe the various risk factors and identify the characteristics of IS in diabetic patients.

## Methods

This was a retrospective, cross-sectional study approved by a local institutional review board. We reviewed the medical records of patients admitted with the diagnosis of acute IS to the Department of Neurology, King Fahd Hospital of the University (KFHU), Alkhobar, Kingdom of Saudi Arabia from February 2010 to December 2015. The study was carried out after getting approval from the institutional review board committee of the university. We retrieved the medical record through the electronic data bank system of the hospital. King Fahd Hospital of the University is a 500 bedded teaching and referral hospital in Alkhobar, Kingdom of Saudi Arabia. Data collection method was the same as mentioned in our previous study on IS.^14^

Stroke was defined according to World Health Organization criteria as a clinical syndrome of a rapidly developing neurological deficit and persisting for more than 24 hours, or leading to death in the absence of another disease that could explain the symptoms. Charts of the patients meeting the diagnostic criteria for stroke were included in the study. All charts were reviewed by a neurologist. Patients with intracerebral hemorrhage, cerebral venous sinus thrombosis, transient ischemic attacks, and cerebral neoplasm were excluded. Patients were divided into diabetic and non-diabetic groups according to presence or absence of diabetes mellitus at the time of presentation. Patients with history of diabetes mellitus before the onset of stroke treated or untreated were included in the diabetic group. Demographic characteristics as age and gender along with presence or absence of other risk factors such as hypertension, hyperlipidemia, ischemic heart disease, atrial fibrillation, smoking and past history of stroke were analyzed.

Hypertension was defined if the patient had systolic blood pressure >140 mm Hg or diastolic blood pressure >90 mm Hg or the patient was on antihypertensive therapy. Ischemic stroke was categorized into 5 major categories according to Trial of Org 10172 in Acute Stroke Treatment classification^15^ as 1) large artery atherosclerosis or macroangiopathic/(LAA), 2) small vessel occlusion or microangiopathic/(SVO), 3) cardio embolic/(CE), 4) stroke of other determined etiology/(OD), 5) stroke of undetermined etiology/ (UDE). Topographic classification of IS was carried out with help of CT scan and MRI brain as total anterior circulation infarction (TACI), partial anterior circulation infarction, LI (lacunar infarction), and posterior circulation infarction. The TACI was again sub classified as anterior cerebral artery and middle cerebral artery (MCA) infarctions. Results of investigations as fasting blood sugar, HbA1c, fasting lipid profile, electrocardiogram (EKG), echocardiogram, carotid ultrasound Doppler and CTA were also collected. Data was entered and analyzed using Statistical Package for the Social Sciences (version 22.0, SPSS Inc, Chicago, IL, USA).

Descriptive statistics as frequencies and percentages were calculated for various major risk factors of IS such as hypertension, dyslipidemia, smoking, ischemic heart disease, atrial fibrillation and past history of stroke. Data was expressed as mean±standard deviation for age and scaled data or median (and interquartile range) for continuous variables and proportion for categorical variables. Chi-square test was used to check proportion between diabetics and non-diabetics for qualitative variables and the limit of statistical significance was set at *p*-value less than 0.05.

## Results

We included records of 127 diabetic patients with IS in our study and compared with an equal number of non-diabetics. Total of 254 records were included. Mean age was 61±13.68 (mean±SD) years for diabetics and 60±16.69 (mean±SD) years for non-diabetics. In the diabetic group, 68.5% were male and 31.5% were female with male to female ratio of >2:1. In the non-diabetic group; 62.2% were male and 37.8% were female with male to female ratio of 1.65:1. Demographic characteristics along with numbers and percentages of risk factors in both groups are presented in **Table 1**. Fasting blood sugar, HbA1c, and fasting lipid profile were checked in all patients. Base line biochemical values are shown in **Table 2**.

**Table 1:** Demographic characteristics and risk factors for ischemic stroke in diabetic and non-diabetic groups.

Risk factors	Diabetic (127)	Non diabetic (127)	*p*-value
Age(mean±SD)	61.67±13.68	60.45±16.69	0.525
Male	87 (68.5)	79 (62.2)	0.178
Female	40 (31.5)	48 (37.8)	0.176
Hypertension	101 (79.5)	96 (75.6)	0.24
Dyslipidemia	86 (68.3)	45 (42.5)	0.000
Ischemic heart disease	35 (28.0)	24 (19.0)	0.167
Past H/O stroke	13 (10.2)	08 (6.2)	0.075
Smoking	09 (7.0)	06 (4.7)	0.52
Atrial fibrillation	12 (9.4)	12 (9.4)	0.254

**Table 2:** Baseline biochemical values for glucose and fasting lipid profile.

Biochemical values	Diabetics	Non diabetics	*P-*value
	(Mean±SD)	
Fasting blood sugar (70-99mg/dl)	174±67.74	105±17.73	0.000
HbA1c (4-6%)	8.96±2.45%	5.86±0.67%	0.000
Cholesterol (≤200mg/dl)	198.87±55.37	184.47±48.41	0.032
Low density lipoprotein/LDL (100-129mg/dl)	128.55±46.19	121.42±43.95	0.222
High density lipoprotein/HDL (35-60 mg/dl)	41.94±15.7	41.97±15.01	0.988
Triglycerides/TG (30-150 mg/dl)	138.33±65.21	115.98±66.98	0.009

The EKG was performed in all patients. Echocardiogram was performed in 109 diabetic and 87 non diabetic patients. In the diabetic group, 8 patients were found to have reduced ejection fraction and 4 had clot. In the non-diabetic group, 6 patients were found to have reduced ejection fraction, 2 had clot, 5 had valvular disease and 1 had cardiomyopathy. Carotid Doppler was carried out in 106 diabetic and 66 non-diabetic patients. The CTA was carried out in selected patients in both groups. Significant extracranial carotid artery disease (>50% stenosis) was detected in 20 diabetics and 14 non-diabetics. 5 diabetics and 6 non-diabetics were found to have significant intracranial stenosis.

**Figure 1:**
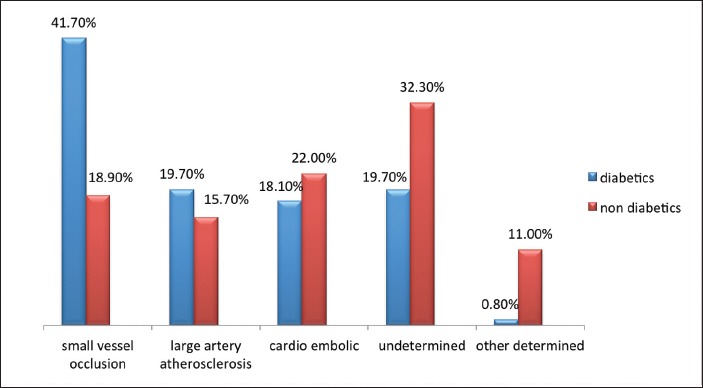
- Etiologic subtypes of ischemic stroke in diabetics and non-diabetics.

Small vessel occlusion was the most common subtype of IS in diabetic patients. It was identified in 53 (41.7%) diabetic patients as compared with 24 (18.9%) non diabetics and was statistically significant (*p*-value=0.000). Stroke of undetermined etiology was the most common subtype in the non-diabetic group, followed by CE and SVO subtypes. Stroke of OD and UDE were significantly more prevalent in the non-diabetic group with *p*-values of 0.000 for OD and 0.016 for UDE. **Figure 1** shows the percentages of different subtypes of IS in both groups.

**Figure 2:**
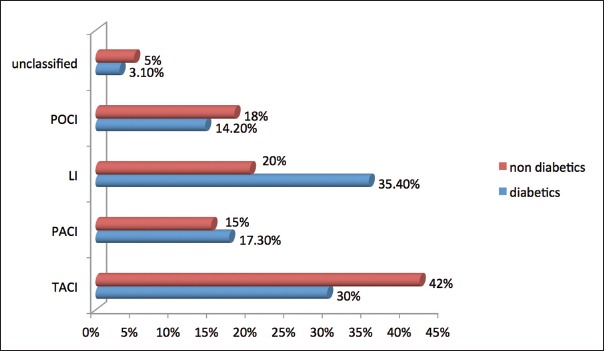
- Topographic localization of ischemic stroke. POCI - posterior circulation infarction, LI - lacunar infarction, PACI - partial anterior circulation infarction, TACI - total anterior circulation infarction

The TACI was the most common finding on neuroimaging in non-diabetics and MCA was the most commonly affected territory. It was significantly more frequent in non-diabetics as compared with diabetics (*p*-value=0.038). Lacunar infarction was the most common finding on neuroimaging in diabetic group and was statistically significant (*p*-value=0.004). **Figure 2** shows the topographic localization of IS on the basis of CT scan or MRI brain in both groups.

## Discussion

The results of our study did not show any statistically significant differences in age and gender distribution between the 2 groups. No significant age difference was identified between the two groups in other studies as well.^5^,^9^,^16^ In one study^17^ diabetics were relatively older than non-diabetics and in a recent multinational study diabetes mellitus was independently associated with a slightly younger age.^18^ A large cohort study from the United Kingdom observed the highest risk for stroke attributable to type 2 diabetes in a relatively young age and particularly in women.^19^

Hypertension, hyperlipidemia^10^,^20^ and ischemic heart disease^20^ were more frequent as compared with other studies. The prevalence of other risk factors in the diabetic group as atrial fibrillation^20^ and past history of stroke^10^ was lower in contrast to other studies. The results of the present study did not show any statistically significant difference in the prevalence of risk factors as hypertension, ischemic heart disease, atrial fibrillation, smoking and past history of stroke between the 2 groups except dyslipidemia. Dyslipidemia was significantly more prevalent in diabetics in our study. This finding is similar to other studies.^10^,^17^,^21^ However, these studies observed hypertension^10^,^17^,^21^ and ischemic heart disease^17^,^21^ also to be more prevalent in the diabetic group in contrast to our study. These findings need to be verified by further large prospective studies. Total cholesterol and triglycerides levels were significantly higher in the diabetic group. This is an important finding to be considered as adequate control of dyslipidemia by life style modification, healthy dietary habits and pharmacotherapy may help in reducing the risk of stroke in diabetic patients. A large prospective hospital based stroke registry concluded that the use of statin before first-ever IS was associated with better outcome and reduced mortality.^22^ A recently published meta-analysis also suggested an association between prestroke statin use and better functional outcome. Interestingly, this association was stronger in patients with small vessel stroke than other stroke subtypes.^23^ As SVO was the most common subtype in the diabetic group, these associations of statin usage and stroke outcome are of great prognostic importance.

Microangiopathic category and LI were more common in the diabetic group than non-diabetic in our study as mentioned in other prospective studies describing the patterns of stroke in diabetic patients.^9^,^10^,^17^ A large prospective European multicenter study conducted by Megherbi et al^9^ characterizing stroke pattern in diabetic patients found that the frequency of intracerebral hemorrhage was lower, the rate of LI was higher, and recovery was worse in diabetic patients. One hospital-based prospective study describing the patients with dysarthria-clumsy hand syndrome reported diabetes in 34% of LI and in 17.8% of non-lacunar stroke (*p*<0.02).^24^ Higher frequencies of LI can be explained by the hypothesis that diabetes mellitus can lead to small vessel arteriolopathy in various organs of the body, especially in the retina, kidneys and brain.^21^

Although there is a lot of published literature from all over the world including the Asian countries on stroke in diabetics, there is no such study from KSA. We need to see whether the diabetic population of KSA have same characteristics or not. This is the first study from KSA describing the characteristics of IS in diabetic patients. The limitation of our study is its retrospective nature; limited sample size and incomplete diagnostic work up in some patients. Carotid Doppler studies were performed in fewer non diabetic patients as compared with diabetics. Incomplete diagnostic work up limits the certainty of etiologic classification of stroke subtypes in both groups. This is a preliminary study from our center which highlights the need of further large, multicenter prospective studies at national levels to estimate the prevalence of significant extracranial as well as intracranial stenosis and other risk factors for IS in diabetic patients of KSA. Furthermore; studying characteristics of IS in Saudi diabetic patients in comparison with non-Saudi patients should also be considered. Diabetes is not only a risk factor for IS but can lead to more insidious brain damage represented by LI and hence increases the risk of dementia as well.^26^ It is the responsibility of health care professionals to increase the awareness among the general population about this important modifiable risk factor for IS.

In conclusion, the results of the current study are comparable to published literature. Dyslipidemia, small vessel occlusive disease, and lacunar infarctions were significantly more prevalent in diabetics than non-diabetics in our study. There is a need to carry out further epidemiological studies to understand the characteristics of IS in diabetics which can help in preventing the first-ever stroke in these patients.
